# High and Far: Biases in the Location of Protected Areas

**DOI:** 10.1371/journal.pone.0008273

**Published:** 2009-12-14

**Authors:** Lucas N. Joppa, Alexander Pfaff

**Affiliations:** 1 Nicholas School of the Environment and Earth Sciences, Duke University, Durham, North Carolina, United States of America; 2 Public Policy, Economics, and Environment, Duke University, Durham, North Carolina, United States of America; Umea University, Sweden

## Abstract

**Background:**

About an eighth of the earth's land surface is in protected areas (hereafter “PAs”), most created during the 20^th^ century. Natural landscapes are critical for species persistence and PAs can play a major role in conservation and in climate policy. Such contributions may be harder than expected to implement if new PAs are constrained to the same kinds of locations that PAs currently occupy.

**Methodology/Principal Findings:**

Quantitatively extending the perception that PAs occupy “rock and ice”, we show that across 147 nations PA networks are biased towards places that are unlikely to face land conversion pressures even in the absence of protection. We test each country's PA network for bias in elevation, slope, distances to roads and cities, and suitability for agriculture. Further, within each country's set of PAs, we also ask if the level of protection is biased in these ways. We find that the significant majority of national PA networks are biased to higher elevations, steeper slopes and greater distances to roads and cities. Also, within a country, PAs with higher protection status are more biased than are the PAs with lower protection statuses.

**Conclusions/Significance:**

In sum, PAs are biased towards where they can least prevent land conversion (even if they offer perfect protection). These globally comprehensive results extend findings from nation-level analyses. They imply that siting rules such as the Convention on Biological Diversity's 2010 Target [to protect 10% of all ecoregions] might raise PA impacts if applied at the country level. In light of the potential for global carbon-based payments for avoided deforestation or REDD, these results suggest that attention to threat could improve outcomes from the creation and management of PAs.

## Introduction

Initiatives to establish new protected areas (PAs) to conserve natural landscapes for species habitat and climate-change mitigation are underway worldwide [1], [2]. Yet, with 13% of global lands already officially under protection, future PA growth is unlikely to even double the extent of the current global PA network or reserve system [3]. Investment in new PAs will need to be efficient, e.g. based upon sophisticated conservation planning tools [4]. However, many current PAs were not created with a systematic eye to achieving conservation priorities [5]. To inform any new investments, here we seek to understand where the past history of PAs has placed protection to date.

Many goals and constraints influenced past PA locations. Given that, have we maximized conservation priorities? To maximize anything, PAs must have impact, i.e. change land-use outcomes. That is, land use would have to differ from what would have occurred in a PA-free world. To change the rate of loss of natural land cover, PAs have to be located where they can prevent forest clearing [6], [7], [8], [9] or other activities that modify natural habitat. Yet the common phrase “rock and ice” summarizes a perception that PA locations are biased towards marginal lands where natural land cover might remain even without a PA. Of course there may be good reasons for such a choice of PA location. In particular, costs of protection could provide an adequate explanation. Often it may be financially and politically expedient to protect land with low financial value [10].

**Figure 1:**
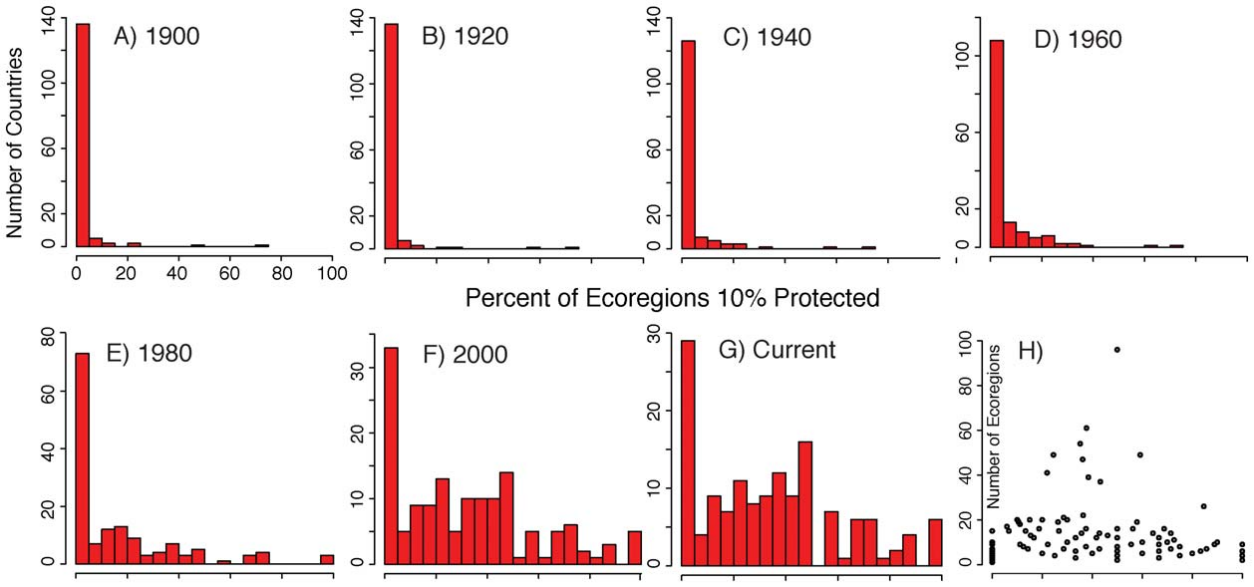
Numbers of countries and the percent of their ecoregions with greater than 10% protection. **A**, **B**, **C**, **D**, **E**, **F**, **G** each plots the cumulative number of countries at 20 year increments. PAs with no date of creation in the database occur first in 1900, and remain in each preceding temporal increment. **H**) Number of ecoregions versus the percent of a country's ecoregions protected. No trend is found for countries with many ecoregions to differ greatly from those with few.

Before explaining such choices or considering changing them, though, we must ask whether the global “rock and ice” perception is correct. Previous national-level studies suggest the adage has a basis in reality [11], [12], [13], [14]. Global studies addressing this issue have also found evidence for PA location bias [1], [2], [15]. An influential example was Hoekstra *et al*.'s [16] results, which showed a clear bias in protection towards certain biomes and ecoregions. These highly protected regions were generally those that receive low levels of land degradation pressure, such as montane grasslands and shrublands. A recent update of global protection shows that this unrepresentative distribution of PAs continues today [3], although each country's coverage continues to evolve (Figure 1).

All of these previous global studies have ignored political boundaries. That is a vital omission. Ecological processes cross borders but most PAs do not. The Convention on Biological Diversity's (CBD) 2010 Target aims to protect 10% of global terrestrial ecoregions [17], for example, but its national policy implications are unclear since many ecoregions cross national borders. Put another way, an often-ignored reality is that the global PA network is composed of many different national networks, all of which have different histories and resulted from a different mix of motivations for conservation. Thus analyzing the distribution of every national PA network, as we do here, provides a large-scale perspective on PA-location biases and it does so at a politically relevant resolution.

We provide the first comprehensive global assessment of the distributions across space of all of the national PA networks of any significant magnitude (>100 km2). We ask whether it is true on the whole, across many countries, that national protection networks have evolved over time to be found disproportionately on higher, steeper, more remote, agriculturally unsuitable lands. We ask too whether the variation in the level of protection across the PAs within a national network is correlated with these indicators of low threat.

## Results

### IUCN Protection vs. No Protection

**Figure 2:**
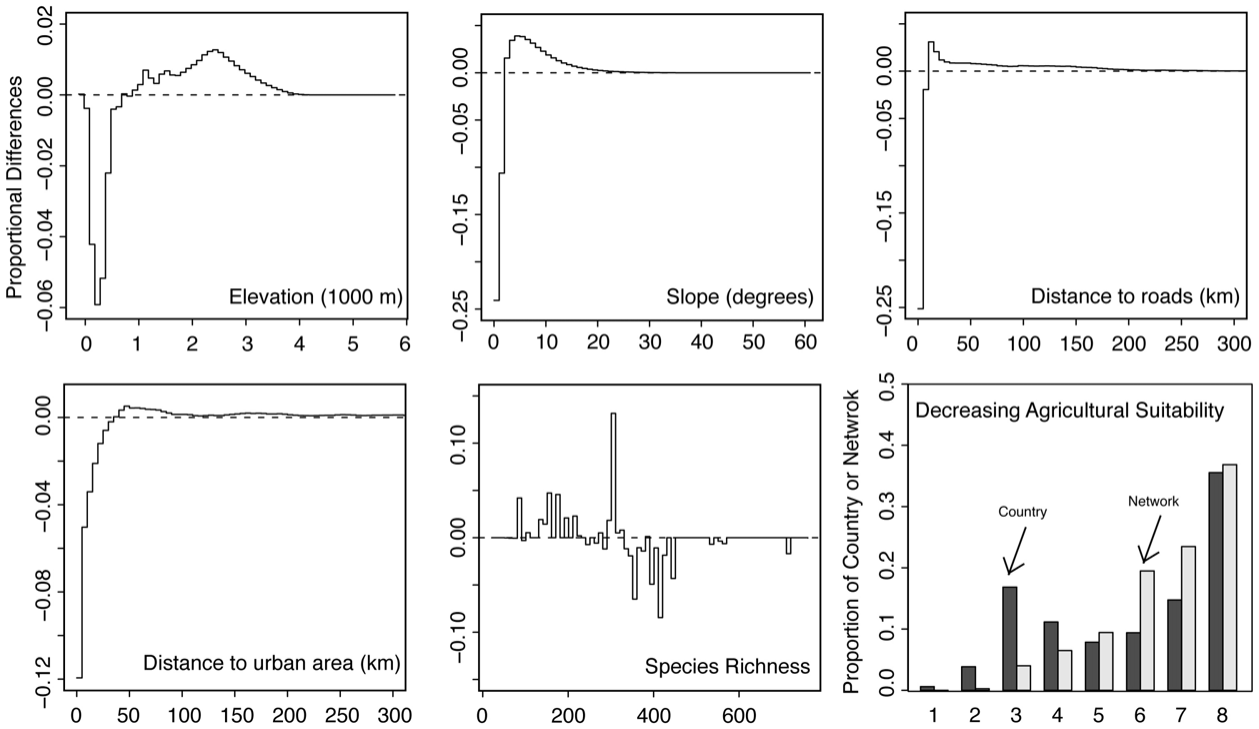
A full description of the IUCN I/VI protected area network for the United States. Variables include elevation, slope, distance to roads, distance to urban areas, species richness, and agricultural suitability. The x-axis corresponds to the variable being measured. The y-axis is the difference between the proportion of the country at each interval and the proportion of the network at the same interval. Values greater than zero (dashed horizontal line) indicate a disproportionate level of protection, while values less than zero indicate a disproportionate absence of protection. Visualizing network distributions in this way allows one to see discrepancies in protection across wide ranges of variables at a high resolution.

First we examine PA distributions within every country that protects 100 km^2^ or more of terrestrial surface (147 countries) and quantify the network's elevation, slope, agricultural suitability, distance to roads, and distance to urban areas, as well as its species richness (see *Materials and* *Methods* for further details on the data and methods used). Then as a first indicator, we calculate the average values for each variable for both the entire country and the entire PA network. Comparing the two indicates whether the network is representative of the country along that dimension. One way to summarize these statistics is demonstrated in Figure 2 for the United States – a country whose network is discussed in an influential paper by Scott *et al*. [12]. It is very clear there that the US has disproportionately protected lands at higher elevations, steeper slopes, lower agricultural suitability, and greater distance from both roads and cities.

For summarizing many countries, additional description is required. Here, we empirically model the probability that a one km^2^ pixel is found within the national PA network as a function of elevation (in meters) [18], slope (in degrees, derived from [18]), distance to roads [19] and urban areas [20] (in kilometers), as well as agricultural suitability [21] (using a 0–9 scale with decreasing rank). Those are all indicators of potential resource extraction or agricultural profit [22]. We also included ecoregion-level species richness [23]. To do this we use a general linear model framework with a probit link (details of the model can be found in *Materials and* *Methods*). If the network consisted of random locations or non-random but representative locations within a country, then such factors would have no power to predict that a pixel is found in the PA network. If, for instance, within any given country a parcel's slope is a significant predictor of that parcel being in the national PA network, then PAs' sites are not representative of the country as a whole.

For all of our variables, we present summaries of such significance across all countries. This lets us summarize global trends in national decisions. For each variable, Table 1a shows the number of countries (of 147) where the variable is a significant predictor of protection. Only countries with significant results are shown (so they need not add to 147). A binomial sign test indicates if the number of significant positives is statistically significantly different from the number of significant negatives; if not, bias is not shown.

**Table 1:** Summary statistics for the individual country models.

	A) Protected vs Unprotected			B) IUCN I/II vs IUCN III/VI		
	Countries	Positive	Negative	Countries	Positive	Negative
**Slope**	132	107**	25	95	67**	28
**Elevation**	140	88**	52	96	64**	32
**Road Distance**	134	108**	26	101	63*	38
**Urban Distance**	140	107**	33	99	60*	39
**Agriculture**	132	76	56	99	53	46
**Richness**	130	50	80*	88	43	45

The left side of the table (**A**) presents the results for models predicting IUCN Category I – VI levels of protection against no protection and corresponds to Figure S1. The right side of the table (**B**) contains results for modeling IUCN Category I or II (most highly protected) within the protected area network and corresponds to Figure S2. The “Countries” column contains the total number of countries where the corresponding predictive variable (ex: slope) was a significant predictive factor of protection. “Positive” indicates the variable was positively correlated with protection, while “Negative” is the opposite. Statistical significance of the trend was conducted using a binomial sign test, and ** indicates the trend is significant at the 0.01 level, while * shows significance at the 0.05 level.

The majority of national networks *are* biased towards higher elevation, steeper slope, and greater distance from roads and cities (Table 1a & Figure S1). The trend for agricultural suitability was not significant across all countries (suitability is in decreasing rank, so that a positive correlation would indicate PAs biased *away* from suitable land). Yet it is in line with other variables as a majority of countries have networks on less suitable lands. A slight majority of national networks is biased away from the species-rich ecosystems.

### Higher vs. Lower Protection

Bias in PA locations could be driven by a particular subset of the network. Six PA-management categories are defined by the International Union for the Conservation of Nature (IUCN). Greater human intervention is permitted as status moves from I to VI. Put another way, lower-numbered categories are designated as more protected. Category I-II PAs tend to be large [24], [25]. Given that characteristic, as well as the lower permitted levels of human intrusion, might they occur on different land than lower category PAs?

We find (Table 1b & Figure S2) that protected areas in IUCN categories I and II, i.e. the more highly protected areas, are on significantly higher and steeper lands further away from roads and urban centers than are IUCN category III – VI protected areas. The results for differences in distance to roads and urban centers are also significant, though less so than the results for slope and elevation. Species richness and agricultural suitability are not significant predictors of the category or status of protection. If these categories imply also higher and lower levels of management effort, then these results suggest that PAs are trying harder where threat is lower. Also, high protection status may be taking credit for a lack of clearing that is due to the undesirability of lands on which it has been placed.

### Using a Summary Index

Table 1 summarizes a number of results for each country across many countries. Further, it does so for two comparisons, protected versus unprotected and higher versus lower protection status. It shows dissimilarities, i.e. that protected lands are significantly different from unprotected lands and higher status lands differ from lower status lands.

Table 1 cannot, though, summarize *how* different protected lands are, in a way that brings together all the differences along the dimensions the table lists (e.g. slope and distance). Further it cannot show whether the difference between protected and unprotected lands is *more or less* than the difference between PAs with higher and lower protection status.

To make those comparisons, we require a single index of similarity across all locations. The impact-evaluation literature regularly employs a probability of receiving intervention [7], [8]. Its analog here, the probability of being in a PA, is useful for us and is provided by the regressions described in Table 1. Those regressions examine the predictors of whether a location is protected or not. Given the results or coefficients from those regressions, one can explicitly predict the likelihood of being protected for each location in a country. That likelihood, the product of location characteristics and the coefficients, is our index.

We find that locations currently within the protected area networks had on average a 24% chance of being protected (keeping in mind that on average only 10% of the country is protected). In contrast, the much larger group of unprotected locations had an 8% chance. Those percentages, however, are unweighted averages across many countries that are quite different in total size (the average country is 8% of the largest country) as well as in network size. When weighting the likelihood-of-protection numbers for each country by its national network size, the protected locations had a 32% chance of being protected while the unprotected had only a 14% chance. Either way, PAs are on lands more than twice as likely to be protected than unprotected lands. That is a considerable difference.

Since weighting clearly matters, we also consider the medians. For protected locations, the median chance of being protected is 21% while for unprotected locations is it 6%, similar results to those above in confirming a significant difference. Looked at another way, looking across all the countries the median ratio of these two probabilities of being protected (for protected versus unprotected locations) is 3.0. By this measure of bias in location, protected lands were three times as likely to be protected as unprotected lands.

Such an index is most useful for comparison purposes. It can be used, for example, to examine whether the differences just discussed are similar to those between higher and lower protection status (about 2/5 of all protected land, on average, is in higher status). Using the same probabilities of being protected, we find that higher status locations had on average a 25% chance of being protected while lower status had a 20% chance (and the network-weighted averages are 27% for high status and 25% for lower). The median probability of protection for high-protection PAs is 18% while the median for lower categorized PAs is 15%. The median of the ratios of these probabilities is 1.3. This is much less bias than when we simply compared all protected versus unprotected lands.

## Discussion

Across 147 countries' national networks, protected areas are indeed non-randomly located on the landscape. The same types of biases also hold, although less so, for the more highly protected area compared with less highly protected areas. All of these results corroborate prior descriptions of specific PA network biases towards, for instance, “rock and ice” [12], [26]. They are also relevant for assessing PAs' conservation impacts.

One area of research where our results are directly relevant is in the quantification of the “conservation success” or effectiveness of protected areas. Interest in this area has been growing rapidly [2], [7], [25], [27], [28], and for good reason. Funders want to learn about the outcomes of their investments, governments want to be maintaining promises to constituents, and conservation biologists want their efforts to be worthwhile. Unfortunately, location bias can confound quantification of impact (see, e.g., Joppa and Pfaff 2009 [6]). A common and reasonable approach to assessing effectiveness is to compare, say, deforestation in the protected area with rates outside [29], [30]. Yet steep slope, large distance to roads and cities and in some instances high elevation and low agricultural suitability can contribute to the inaccessibility of a landscape and thus lower the probability that land is deforested [22], [31]. Here we show these factors to be overwhelmingly associated with protection. This can result in claiming land cover impacts for protection that are actually due to a PA network's landscape characteristics.

This same concern applies to the differing impacts across PA management categories. Location bias means that much of the observed habitat retention in PA Categories I and II [25] could be due to land characteristics: PAs in general are on less threatened land and highly categorized PAs are on a less-threatened subset. It is worth noting, then, that much of the growth in the global PA network has been in categories III-VI [5], [24]. We show that those are closer to threats. That could raise impact. Yet such locations are typically allocated less management effort, to this point, for strict biodiversity conservation [24].

Such results also naturally raise the question of why some PA networks are representative while others are not. An exploratory examination finds no impact of total population or density of population or GDP on the sign and magnitude of coefficients reported above. What this likely means is that the issue of where protection is located is too variable, even within a country or region, to be easily summarized at a global scale. For example, PAs within the United States are (on average) preferentially located on marginal lands [32]. Even so, the National Wildlife Refuge System (NWRS) targets breeding-bird populations with certain habitat characteristics and is preferentially located on lower elevation lands with higher productivity and soil quality [33]. Thus not only is the global network composed of the national networks, which have differing dynamics, but also a national network can have multiple components that operate differently. This highlights the fact that a more context-specific examination of PA-location choice will often be required to fully inform policy.

Fortunately, many recent PAs have been (and others will be) created under consistent and locally contextualized frameworks. Systematic conservation planning [34] is a rapidly growing discipline and is increasingly sophisticated in its incorporation of many factors such as both multiple ecosystem service densities [35] and costs [34], [36]. Algorithms employed in systematic conservation planning are already capable of maximizing the protection of highly threatened landscapes, and can handle the necessary trade-offs between such variables as land procurement costs and number of species protected or amount of threat abated [34]. Indeed, it is now common to claim that PAs need to be in systematically chosen places [37]. Along these lines, our objective in this study is to provide evidence that the national-level bias within PA networks is a global phenomenon.

Our results support the idea that targeting and blocking threat may deserve higher priority in the future creation and management of PAs. The issue of locating protection in areas of greatest threat has received the most attention in the discussion of biodiversity hotspots [38] and other global prioritization schemes [39]. Biodiversity hotspots are regions around the world with high species richness and correspondingly high levels of habitat destruction. Myers *et al*. [38] argue that placing effective protection in these regions is a logical way to protect significant numbers of species that, in the absence of protection, would likely be lost. We agree with this concept, and our results highlight the increasing realization that future PA allocation must differ from historic protection strategies.

## Methods

All datasets are global in scale, in raster (grid) format, and projected into Albers Equal Area projection at a one km^2^ resolution. We used ArcGIS 9.1 to harmonize projections, cell size, and extent and used Python 2.4 in order to remove all marine areas and to create individual text files for each variable for each country. All further analyses were done in R 2.7.1 [40].

The analytical framework we used was a general linear model (package “glm” in R 2.7.1) with a probit link because the regressions we run here involve binary outcomes. In the first set of regressions (for Table 1a), we are explaining whether or not a location is in the nation's protected area network. In the second set of regressions (for Table 1b), we are examining only the locations that are in the network and explaining whether or not a location is in a protected area that is accorded higher status. One important point is that the coefficients for a given variable not only need not be the same in those regressions and could even be different in sign (for instance, protection may be biased towards steep slopes but, within the protected network, higher status could be biased towards flat areas). Thus Table 1a and 1B do not have similar results by construction. These probabilities were generated using the “predict” command, along with the coefficients from the original regression models, in the “stats” package in R 2.7.1.

Information on PA location came from the 2007 World Database on Protected Areas (WDPA) [41]. Only PAs classified by the International Union for Conservation of Nature (IUCN) in categories 1 through 6, and only countries with PA networks of 100 km^2^ or more, were included. When two PAs overlapped, we assigned that area the highest IUCN classification of the two. Due to high potential error rates [3], PAs without polygon boundaries (i.e., point representation only) were not included.

For comparisons across different management categories, we included only countries with 100 km^2^ or more of categories I – II *and* 100 km^2^ or more of categories III – VI. To analyze protection over time (Figure 1), we used information included in the WDPA on date of PA creation. When analyzing over time, PAs with no date were included in each temporal step, distributing the error uniformly over the analysis.

We obtained elevation data from the Shuttle Radar Topography Mission (SRTM) [18]. The source for this data set was the Global Land Cover Facility (www.landcover.org). The SRTM gathered elevation data on a near-global scale, generating a very complete high-resolution elevation database. We calculated slope values from the SRTM elevation dataset. All slope values are degrees from horizontal. Distance to roads was calculated from a vector road network extracted from the VMAP Level0 dataset [19]. While the quality of this data is variable it is the only freely available global road dataset to characterize the global road network. Distance to urban areas was calculated using the Gridded Rural Urban Population dataset (GRUMP) [20], which provides a gridded and global extent of urban population. Agricultural suitability was taken from a dataset provided by the International Institute for Applied Systems Analysis [21]. The dataset (plate 28) incorporates climate, soil type, land cover, and slope of terrain to measure agricultural suitability, ranking each grid cell from 0 (no constraints) to 9 (severe constraints). We used the World Wide Fund for Nature (WWF) Ecoregions product to determine ecoregion type [23]. The Ecoregions product delineates 8 biogeographic realms, 14 biomes, and 867 ecoregions. Included in the WWF Ecoregions project are data on terrestrial vertebrate species richness. These data encompass more than 26,000 terrestrial vertebrates (amphibians, reptiles, birds, and mammals) and were gathered from literature, expert opinion, and online datasets. Richness is at the resolution of ecoregion.

## Supporting Information

Figure S1 Global maps of predictors of all categories of protection for elevation, slope, distance to roads, distance to urban areas, agricultural suitability, and species richness. Red indicates that the variable was a significant and positive factor in a regression model explaining protection. Yellow shows a significant and negative association, black indicates the variable was not a significant predictor for that country, while grey shows those countries with less than 100 km^2^ of protected area. See Table 1a in the main text for the summary statistics attached to these results.
(3.01 MB TIF)

Figure S2 Global maps of predictors of IUCN Category I or II protection within the entire protected area network for a country elevation, slope, distance to roads, distance to urban areas, agricultural suitability, and species richness. Red indicates that the variable was a significant and positive factor in a regression model explaining protection. Yellow shows a significant and negative association, black indicates the variable was not a significant predictor for that country, while grey shows those countries with less than 100 km^2^ of protected area. See Table 1b in the main text for the summary statistics attached to these results.
(2.93 MB TIF)
