# Supplementary figures and images for: High and Far: Biases in the Location of Protected Areas
Source: PLoS One. 2009 Dec 14;4(12):e8273. doi: 10.1371/journal.pone.0008273 (PMC2788247; doi:10.1371/journal.pone.0008273)

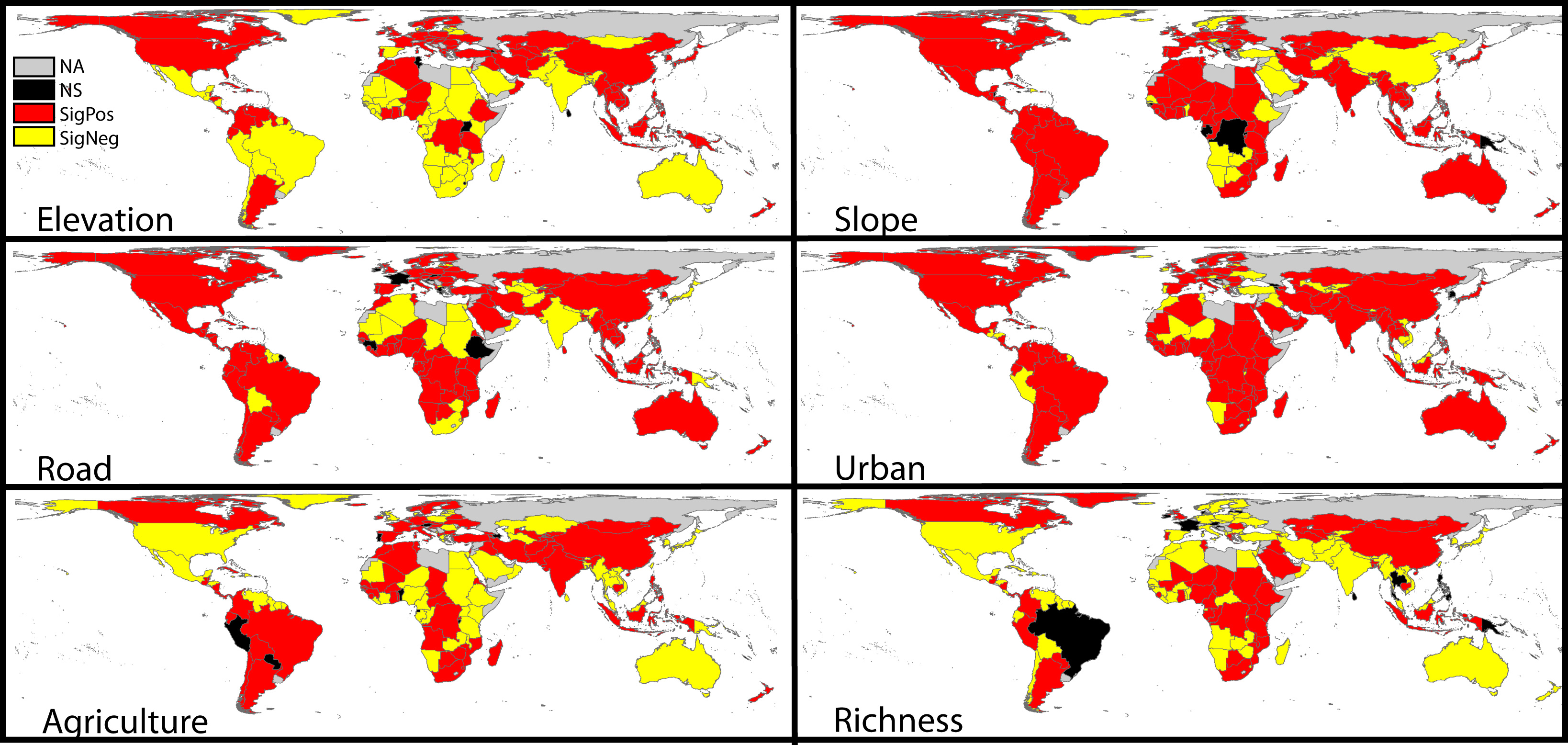

Supplement: Figure S1 — Global maps of predictors of all categories of protection for elevation, slope, distance to roads, distance to urban areas, agricultural suitability, and species richness. Red indicates that the variable was a significant and positive factor in a regression model explaining protection. Yellow shows a significant and negative association, black indicates the variable was not a significant predictor for that country, while grey shows those countries with less than 100 km2 of protected area. See Table 1a in the main text for the summary statistics attached to these results. (3.01 MB TIF) [file pone.0008273.s001.tif]

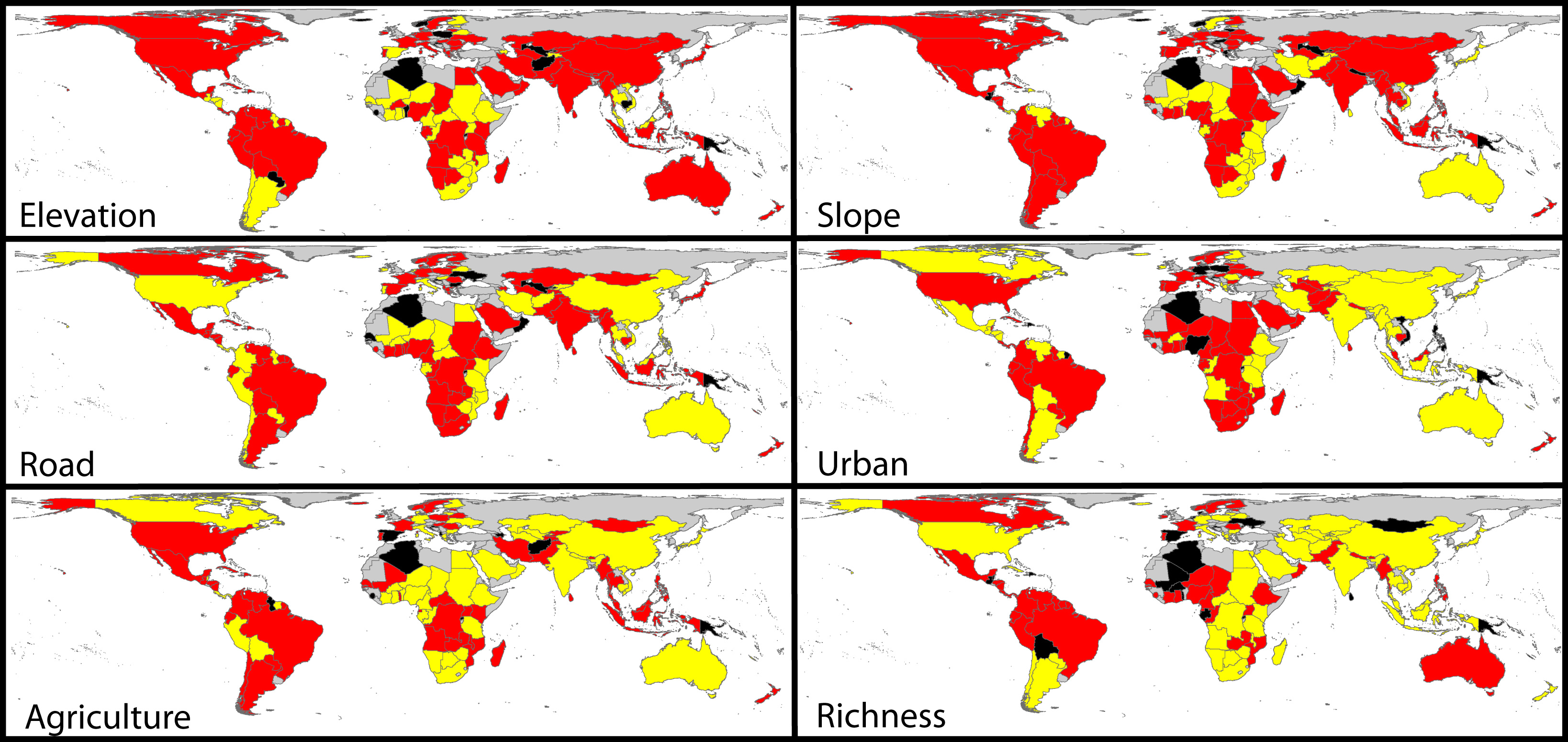

Supplement: Figure S2 — Global maps of predictors of IUCN Category I or II protection within the entire protected area network for a country elevation, slope, distance to roads, distance to urban areas, agricultural suitability, and species richness. Red indicates that the variable was a significant and positive factor in a regression model explaining protection. Yellow shows a significant and negative association, black indicates the variable was not a significant predictor for that country, while grey shows those countries with less than 100 km2 of protected area. See Table 1b in the main text for the summary statistics attached to these results. (2.93 MB TIF) [file pone.0008273.s002.tif]
